# Systematic review and clinical recommendations for dosage of supported home-based standing programs for adults with stroke, spinal cord injury and other neurological conditions

**DOI:** 10.1186/s12891-015-0813-x

**Published:** 2015-11-17

**Authors:** Ginny Paleg, Roslyn Livingstone

**Affiliations:** Montgomery County Infants and Toddlers Program, Rockville, MD USA; Sunny Hill Health Centre for Children, Vancouver, British Columbia Canada

**Keywords:** Standing frame, Supported standing, Range of motion, Standing balance, Standing devices, Stander, Tilt-table

## Abstract

**Background:**

Sitting for more than 8 h a day has been shown to negatively impact health and mortality while standing is the recommended healthier alternative. Home-based standing programs are commonly recommended for adults who cannot stand and/or walk independently. The aim of this systematic review is to review effectiveness of home-based standing programs for adults with neurological conditions including stroke and spinal cord injury; and to provide dosage guidelines to address body structure and function, activity and participation outcomes.

**Methods:**

Eight electronic databases were searched, including Cochrane Library databases, MEDLINE, CINAHL and EMBASE. From 376 articles, 36 studies addressing impact of a standing intervention on adults with sub-acute or chronic neurological conditions and published between 1980 and September 2015 were included. Two reviewers independently screened titles, reviewed abstracts, evaluated full-text articles and rated quality and strength of evidence. Evidence level was rated using Oxford Centre for Evidence Based Medicine Levels and quality evaluated using a domain-based risk-of-bias rating. Outcomes were divided according to ICF components, diagnoses and dosage amounts from individual studies. GRADE and the Evidence-Alert Traffic-Lighting system were used to determine strength of recommendation and adjusted in accordance with risk-of-bias rating.

**Results:**

Stronger evidence supports the impact of home-based supported standing programs on range of motion and activity, primarily for individuals with stroke or spinal cord injury while mixed evidence supports impact on bone mineral density. Evidence for other outcomes and populations is weak or very weak.

**Conclusions:**

Standing should occur 30 min 5 times a week for a positive impact on most outcomes while 60 min daily is suggested for mental function and bone mineral density.

**Electronic supplementary material:**

The online version of this article (doi:10.1186/s12891-015-0813-x) contains supplementary material, which is available to authorized users.

## Background

Sitting for more than 8 h per day has been shown to increase mortality [1] while standing is a healthier alternative that can positively affect mortality in adults [2, 3]. Adults who are non-ambulatory due to neurological conditions such as stroke, spinal cord injury (SCI), acquired or traumatic brain injury or multiple sclerosis (MS) often sit for more than 8 h a day, and as a result, experience painful, problematic and costly secondary complications [4]. These include body structure and function impairments [5] such as altered muscle tone or spasticity, range of motion (ROM) limitations or contractures, muscle weakness, constipation, decreased bone mineral density (BMD) with increased risk for fractures and bone pain, as well as activity limitations and participation restrictions. These may be related to long-term sitting and lying postures in those with chronic conditions but also impact individuals in the sub-acute phase a few weeks after onset of disease or injury [6–11].

Supported standing devices such as standers, tilt-tables or standing wheelchairs allow the user to attain and maintain a standing or partial-standing position and commonly stabilize hips, knees and ankles through posterior heel, anterior knee and posterior hip supports and/or straps. A systematic review [12] supported the beneficial effects of standing devices on BMD, ROM, spasticity, and bowel function for participants of all ages with neurological dysfunction. A systematic review of the impact on ROM, spasticity, BMD and activity outcomes only [13], concluded that supported standing may prevent small losses of ankle mobility and that long-term, higher dose programs may slow bone loss.

Supported standing programs have been integrated into clinical practice for over 50 years [14–19] and yet, there are no published evidence-based guidelines defining how long or how often adults with neurological conditions need to stand to effect change in body structure and function, activity or participation outcomes. Given that standing equipment can be expensive [20] and personnel costs and time to assist with use [21] (as reported in Walter et al.,[22]) have a potentially significant impact on health economic resources; it is essential that the evidence supporting outcomes of standing programs should be established. The aim of this systematic review is to evaluate the evidence for all outcomes potentially impacted by a supported standing program in adults with chronic neurological conditions. The primary aim is to establish evidence of effectiveness, with a secondary goal being to identify evidence-based dosage recommendations for home-based programs.

## Methods

The Preferred Reporting Items for Systematic Reviews and Meta-Analyses (PRISMA) [23] statement was used to structure this review. Electronic databases were searched from 1980 to September 2015 and included: EBM Reviews: Cochrane Central Register of Controlled Trials, Cochrane Database of Systematic Reviews, Database of Abstracts of Reviews of Effects (DARE), ACP Journal Club; CINAHL; Medline and EMBASE. Search terms included ‘standing’, ‘tilt-table’, ‘standing frame’, ‘standing position’, ‘standing equipment’, ‘stander’, ‘standing wheelchair’ and ‘supported standing’. No limits were placed on design methodology, language or publication status in the initial search. See Additional file 1 for details.

Bibliographies of electronically retrieved studies and review articles were manually searched to identify additional publications. Both authors independently read all titles and abstracts and agreed on articles to be retrieved full text. Following independent full-text review, both agreed to studies meeting inclusion criteria. Differences of opinion were resolved at all stages through discussion and consensus without the need to involve a third reviewer.

The initial search included all primary source studies including adults aged 19 years or older, with a neurological diagnosis, participating in a supported standing intervention. A stander was defined as a device that stabilized the hips, knees and ankles. A standing intervention was defined as being positioned above 60° (from horizontal) for at least 10 min for a minimum of five sessions within a 2-week period. Studies that used additional interventions such as functional electrical stimulation or whole body vibration were excluded unless there was also a supported standing only phase in the study. Studies where participants engaged in only one or two sessions of standing in total, or that were primarily investigating physiological responses to being tilted from supine to upright in less than 10 min were excluded. Patients in the acute phase immediately following onset or injury have different considerations to those able to engage in active rehabilitation or with chronic conditions, and those populations were excluded. To meet inclusion criteria, studies needed to be published in English, in a peer-reviewed journal and provide clear information on standing dosage.

Data were extracted independently by both authors, and consensus on content of tables and ratings achieved through discussion. Quality assessment of Evidence Level 1–4 studies was completed using a domain-based risk-of-bias approach [24]. Domains were rated as low, moderate, serious or unclear-risk with the lowest score used as the overall rating for individual studies (Additional file 2). Level 5 studies were not rated as most criteria were inappropriate and evidence lower quality.

Outcomes were divided into International Classification of Functioning (ICF) [5] components of body structure and function, activity and participation. To evaluate dosage, body structure and function was divided into categories. Standing balance, gait, transfers and self-care were included under activity and participation. While vestibular reactions are considered to be body structure and function, maintaining a body position such as standing is coded under activity in the ICF [5]. Quality of life was included under mental function as evidence of subjective sense of well-being. Level of evidence was rated using Oxford Centre for Evidence Based Medicine Levels [25]. Single-subject research designs are not included in this rating system but those with at least three intervention/withdrawal phases and appropriate visual analysis of data were rated at Oxford level 4. Strength of recommendation was rated using Grading of Recommendations, Assessment, Development and Evaluation working group (GRADE) guidelines [26] and the Evidence Alert Traffic-Lighting System [27]. Strong GRADE [26] recommendations lead to a Green traffic-lighting code indicating that high-quality evidence supports use of this intervention. Weak ratings lead to a Yellow traffic-lighting code indicating evidence is weak or inconclusive and that clinicians should measure outcomes. Red traffic-lighting codes indicate that strong evidence demonstrates that the intervention is ineffective.

## Results

**Fig. 1:**
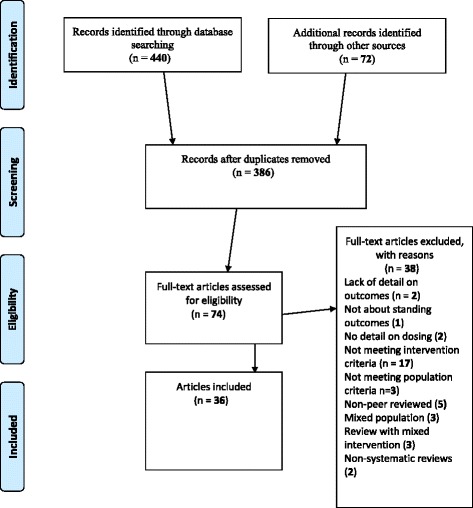
PRISMA flow diagram of the search results

The PRISMA [23] flowchart outlining each step is shown in Fig. 1. The electronic database search strategy identified 440 titles with an additional 72 titles identified through manual searching. Following duplicate removal, 386 titles remained and 74 titles were retrieved full text. Following full-text review, 36 articles met inclusion criteria [13, 20, 22, 28–60] with 95 % initial agreement between reviewers. One systematic review [13] met inclusion criteria for population and intervention but provided no specific dosage recommendations. Although the exclusion of non-peer reviewed literature could raise concerns about publication bias, this primarily involved additional single-case study [61, 62] or survey data [21, 63]. One group study [64] suggesting positive benefit on pulmonary function for sub-acute SCI was only available as an abstract in conference proceedings and did not provide sufficient detail for inclusion. See Additional file 3 for details of excluded studies. Table 1 lists characteristics of included primary research articles with study design, population and intervention characteristics, results and risk-of-bias [24] summary scores.

**Table 1:** Characteristics of included primary studies

Study	Design	Risk of bias (Quality)	*N*	Popu-lation	Standing intervention	Time	Results
Adams & Hicks 2011 [28]	Randomized crossover	Serious risk (low quality)	7	Chronic SCI	Tilt table angle 68.6° ± 11.3	45 mins, 3 × wk × 4 weeks (135 mins/week)	Extensor spasms were reduced to a greater degree with standing than BWSTT. Flexor spasms, clonus, self-reported mobility, and QOL tended to benefit more from 4 weeks of BWSTT than standing alone
Alekna et al. 2008 [29]	Longitudinal	Serious risk (low quality)	54 total–27 matched pairs	Sub-acute SCI followed for 18 mos	Upright standing frame	60 mins, 5 × wk (300 mins/week)	No SS difference between standing and non-standing groups in yr 1. After 2 years those standing >/=1 h daily, 5 days/week had SS higher leg BMD than non-standing group
Allison & Dennett 2007 [30]	RCT	Moderate risk (moderate quality)	17	Sub-acute stroke	Upright stander	45 mins, 5 × wk (225 mins/week)	Intervention group: SS improvement Berg Balance score between wk 1 and 12, in intervention group. Non SS higher scores on all motor measures wk 12
Bagley et al. 2005 [31]	RCT	Moderate risk (moderate quality)	167 total–71 intervention, 69 controls	Sub-acute stroke	Upright standing frame	26 mins × 14 sessions (182 mins/week)	No SS difference between groups on any outcome measure or decrease in resource use
Baker et al. 2007 [32]	Randomized crossover	Moderate risk (moderate quality)	6	Chronic MS	Upright standing frame	30 mins daily × 3 weeks (210 mins/week)	SS improvement in hip and ankle ROM in standing vs exercise phase for both groups. No SS differences in spasticity or spasm although downward trend seen
Ben et al. 2001 [33]	RCT	Moderate risk (moderate quality)	20	Sub-acute SCI	Tilt-table, vertical	30 mins, 3× wk × 12 weeks (90 mins/week)	Mean treatment effect on ankle ROM of 4° and on femur BMD of 0.005
Bohannon & Larkin 1985 [34]	Case series	Moderate risk (moderate quality)	20	Sub-acute and chronic SCI	Tilt table, 70°	30 mins × 2.3–6.4 × wk (69–192 mins/week)	Passive ankle dorsiflexion ROM increases in all subjects of between 3 and 17° at a calculated rate of 0.11 to 1.0° a day
Bohannon 1993 [35]	Case study	N/A (low quality)	1	Chronic SCI	Tilt table, 80°	30 mins × 5 sessions (150 mins/week)	Each day’s standing trial followed by immediate reduction in lower extremity spasticity (modified Ashworth scale and pendulum testing). Spasms reduced until following morning-helpful for performance of car transfers
Cotie et al. 2010 [36]	Randomized crossover	Moderate risk (moderate quality)	7	Chronic SCI	Tilt table, to maximum angle tolerated or 80°	30 mins, 3 × wk × 4 weeks (90 mins/week)	Resting skin temperature decreased at 4 sites after 4 weeks BWSTT. Resting skin temp decreased at right thigh only after 4 weeks standing. Both BWSTT and standing training altered reactivity of skin temperature at all sites except the right calf following single session. 1 session BWSTT skin temperature decreased at 6 sites
							I session standing skin temperature decreased 2/6 sites
De Bruin et al. 1999 [37]	SSRD—MBD	Serious risk (low quality)	19	Sub-acute SCI	Upright stander	60 mins, 5 × wk (300 mins/week)	Marked decrease trabecular bone in the nonintervention subjects. Subjects beginning standing program early showed no or insignificant loss of trabecular bone
Dunn et al. 1998 [38]	Cross-sectional survey	N/A (low quality)	99	Chronic SCI	Upright stander	30–60 min 1–6×/week (30–360 mins/week)	Less than 10 % experienced side effects e.g. nausea or headaches 21 % reported being able to empty their bladder more completely. Favorable response on effects of the standing on bowel regularity, reduction of urinary tract infections, leg spasticity, and number of bed sores. 79 % of subjects highly recommended standing devices
Edwards & Layne 2007 [39]	Case series	Serious risk (low quality)	4	Chronic SCI	Upright stander	60 mins, 2 × wk × 12 weeks (120 mins/week)	Subjects actively responded to exercise in the standing device, as measured by EMG, HR, and BP
Eng et al. 2001 [20]	Cross-sectional survey	N/A (low quality)	126	Chronic SCI	Upright stander or walker and long-leg braces	40 min 3.8 ×/week (152 mins/week)	Reported improved well-being, circulation, self-care, skin integrity, reflex activity, bowel and bladder function, digestion, sleep, pain, and fatigue. The most common reason preventing respondents from standing was cost of standing equipment
Eser et al. 2003 [40]	RCT	Serious risk (low quality)	38 (19 in each group)	Sub-acute SCI	Passive standing angle or device not stated	30 mins, 5 × wk (150 mins/week)	No SS difference between 30 mins FES cycling or 30 mins standing. Tibial cortical BMD decreased by 0–10 % of initial values within 3–10 mos. Mean decrease BMD 0.3 % per month FES group and 0.7 % in standing group
Frey-Rindova et al. 2000 [41]	Longitudinal	Serious risk (low quality)	29	Sub-acute SCI followed for 2 years	Upright stander	30 mins, 3 × wk (90 mins/week)	12 mos after SCI: tetraplegic - SS decrease BMD in trabecular bone of radius and tibia; paraplegic - decrease in tibia BMD only. No SS influence of physical activity intensity. Tilt table standing in early rehabilitation may attenuate decrease of BMD of tibia in some
Goemaere & Laere 1994 [42]	Cross-sectional	Moderate risk (moderate quality)	53	Chronic SCI	Upright stander	60 mins, 3–7 × wk × 52 weeks (180–420 mins/week)	Standing group better-preserved BMD at femoral shaft (p = 0.009), but not at proximal femur, than non-standing. BMD at lumbar spine (L3, L4) marginally higher in standing group (SS only for L3). Subgroup standing with long leg braces SS higher BMD at proximal femur than those using a standing frame or wheelchair
Goktepe et al. 2008 [43]	Cross-sectional	Moderate risk (moderate quality)	71	Chronic SCI	Upright stander	60 mins daily (420 mins/week)	No SS difference in BMD found among mean t-scores of lumbar and proximal femoral regions of those standing > 1 h, < 1 h or non-standing. Standing >1 h daily -slight tendency to higher t-scores
Hoenig & Murphy 2001 [44]	Case study	N/A (low quality)	1	Chronic SCI	Upright stander	60 min 5 × wk (300 min/week)	Significant increase in frequency of bowel movements and decrease in bowel care time with use of standing table 5 times/week vs baseline
Kim et al. 2015 [57]	RCT	Moderate risk (moderate quality)	30	Sub-acute stroke	Tilt table (subjects determined angle)	20 min 5×/week (100 min)	SS increase in the strength of all LE muscle groups, gait velocity, cadence, stride length, decrease in double limb support period, and improvement in gait symmetry in task-oriented training on a tilt table group vs standing only or standing on 1 leg only groups.
Kim et al. 2015 [58]	RCT	Moderate risk (moderate quality)	39	Sub-acute Stroke	Tilt table (subjects determined angle)	20 min 5×/week (100 min/week)	SS increase in EMG patterns of affected leg extensors and flexors and clinical scores in standing with task-oriented training group vs controls or standing alone. SS improvement in functional status and lower extremity movement in tilt table standing group vs controls
Kunkel et al. 1993 [45]	Case series	Serious risk (low quality)	6 (4 SCI, 2 MS)	Chronic SCI and MS	Upright stander	144 h over 135 days = 64 mins day × 7 (448 mins/week)	No important differences between initial and final scores for clinical assessment and ROM. 3 subjects for whom H-reflexes were found, latency and amplitude not altered by standing. BMD normal in lumbar spine but sig reduced in femoral neck. Standing did not modify BMD in any site. 67 % of subjects continued to “stand” and felt healthier because of it
Kuznetsov et al. 2013 [46]	RCT	Serious risk (low quality)	104 divided between 3 groups	Sub-acute stroke	31 controls used tilt table, 60°–80°	20–30 mins day × 30 days (140–210 mins/week)	Compared robotic tilt-table training (ROBO) plus FES vs ROBO vs tilt-table only (controls). 8 controls prematurely quit study due to orthostatic reactions. BP and cerebral blood flow dipped <10 % during ROBO. 52 % of controls - mean arterial pressure decreased by ≥20 %. ROBO-FES increased leg strength by 1.97 ± 0.88 points, ROBO by 1.50 ± 0.85 more than controls (1.03 ± 0.61, P < 0.05). Cerebral blood flow volume increased in ROBO groups more than controls (P < 0.05)
Kwok 2015 [59]	Randomized Crossover	Moderate quality (moderate risk)	17	Chronic SCI	Tilt-table, as upright as possible	30 mins day 5 × wk × 6 weeks (150 mins/week)	No difference in time to first stool or time for bowel care routine. 8/17 reported improved bowel function including decreased abdominal distention. Some participants reported decreased muscle tone, improved posture in wheelchair and sense of achievement.
Lee et al. 1996 [47]	RCT	Serious risk (low quality)	60	Sub-acute stroke	Upright stander with/without biofeedback	20 mins day × 2–4 weeks (140 mins/week)	SS improvement in static standing steadiness (p < 0.002) in group using biofeedback
Matjacic et al. 2003 [48]	Case study	N/A (low quality)	1	Chronic stroke	Upright dynamic stander	20 mins, 10 sessions (100 mins/week)	Subject demonstrated substantial functional improvement and improved weight-shifting ability following 10 days balance training in a specialized standing frame with computer feedback
Nelson & Schau 1997 [49]	SSRD	Low risk (high quality)	1	Chronic CP	Upright stander	Work day	Small increase in work output when positioned in the standing table but dramatically improved posture
Netz et al. 2007 [50]	Case series	Low risk (high quality)	13	Chronic - various	Upright standing box	Mean 16 mins, 47 sessions in 12 weeks. (62 mins/week)	Significant post-intervention improvements in LE muscle strength. Improvements measured with FIM in sphincter control, locomotion, mobility, motor score, and total score. Over 60 % of those previously requiring assistance to stand were able to stand for an average of 1 min unassisted and walk an average of 14 m with a walker
Odeen & Knutsson 1981 [51]	Case series	Serious risk (low quality)	9	Chronic SCI	Tilt-table 85° Feet in 15° dorsal or plantar flexion	30 mins × 8 sessions, 4 consecutive days (120–210 mins/week)	Following weight-bearing stretch in standing with feet in dorsal or plantar flexion, average reduction in resistance to passive movement was 32 % and 26 %, respectively. Following un-weighted stretch in supine, average reduction was 17 %
Richardson 1991 [52]	SSRD	Serious risk (low quality)	1	Sub-acute TBI	Upright stander	10 mins daily × 7 days (70 mins/week)	Subject increased tolerance for standing and ankle ROM increased
Robinson et al. 2008 [53]	RCT	Low risk (high quality)	30	Sub-acute stroke	Upright stander	30–40 mins × 5 days a wk × 4 weeks (150–200 mins/week)	Same ankle ROM at 4 and 10 week for 2 interventions: splint with affected ankle plantargrade, 7 nights wk vs tilt table standing with ankle at maximum dorsiflexion, 5 × wk
Shields & Dudley-Javoroski 2005 [54]	Case study	N/A (low quality)	1	Chronic SCI	Standing wheelchair	30–40 mins × 5 days a wk (130.4 mins/week)	Data-logger indicated client chose to stand for multiple short bouts (mean = 11.57 min) at average angle of 61° and average of 3.86 ×/week. He achieved 130.4 % of goal (20 mins 5 ×/week) resulting in average of 130.4 min/week. Subjective reports of improved spasticity and bowel motility
Singer et al. 2004 [55]	Longitudinal	Unclear risk (low quality)	105	Acute TBI	Upright stander	30 mins daily (210 mins/week)	Ankle contracture identified in 40/105 patients studied. In 23/40 contracture resolved with PT including prolonged weight-bearing stretches. 17/40 contracture worsened. 10/17 required serial plaster casting (+/− injection of botulinum toxin type A). Remediation of ankle contracture not a priority in 7/40 due to disability severity. Dystonic extensor muscle over-activity major contributor to persistent or progressive ankle contracture
Taveggio et al. 2015 [60]	RCT	Moderate risk (moderate quality)	8	Sub-acute ABI in VS or MCS	Tilt table at 65°	30 mins 3×/week × 24 sessions	Robotic stepping reduced cardiovascular distress in 3 out of 4 patients. Orthostatic hypotension worsened in 3 out of 4 patients in the static standing only group
Walter et al. 1999 [22]	Cross-sectional Survey	N/A (low quality)	99	Chronic SCI	Upright stander	>30 min 7×/week (>210 mins/week)	Respondents (n = 99) who stood ≥30 min/day had sig improved QOL, fewer bed sores, fewer bladder infections, improved bowel regularity, and improved ability to straighten their legs compared with those who stood less time. Compliance with regular home standing (at least once per week) was high (74 %)
Wong & Lee 1997 [56]	RCT	Serious risk (low quality)	60	Sub-acute stroke and TBI	Upright stander with/without biofeedback	60 mins 5 × wk × 2–4 weeks (300 mins/week)	After 4 weeks, % postural asymmetry in intervention (with biofeedback) and controls was reduced from 17.2 +/− 10.8 % and 17.0 +/− 10.0 % to 3.5 +/− 2.2 % and 10.1 +/− 6.4%, respectively (p = 0.003)

Outcomes were divided into ICF [5] components with details reported below. Quality of evidence and strength of recommendation for each outcome are reported along with suggested dosage recommendations in Table 2.

**Table 2:** Evidence strength and dosage suggestions divided according to population within ICF components

					Grade			
ICF	Population	Citation	Pertinent results	Oxford level	Evidence quality	Recommendation	Traffic light	Suggested dosage
Range of motion	SCI	Ben [33]	4° increase ankle ROM	2	Moderate	Strong	Green - GO	30 mins 5 × wk
		Bohannon [34]	8° increase ankle ROM	4				
		Dunn [38]	Increased ability to straighten legs	5				
		Walter [22]	Increased ability to straighten legs	5				
	Other - MS	Baker [32]	SS increase hip and ankle ROM	3	Low	Weak +	Yellow- Measure	30 mins daily
	Stroke	Robinson [53]	Maintained ankle ROM	2	High	Strong	Green - GO	30 mins 5 × wk
	TBI/ABI	Richardson [52]	Decreased ankle contracture	4	Unclear	Weak +	Yellow-Measure	30 mins 5 × wk
		Singer [55]	Eliminated ankle contracture	4				
Activity (Balance, mobility, transfers, ADL)	Stroke	Allison [30]	SS difference Berg Balance Scale (p < 0.05)	2	Low to Moderate	Strong	Green – GO	30 mins 5 × wk
		Kim [58]	SS improvement in functional abilities and lower limb movement (p < 0.01)	2				
		Lee [47]	SS increased standing steadiness (p < 0.02)	2				
		Wong [56]	SS increased postural symmetry (p < 0.003)	2				
		Matjacic [48]	Increased weightbearing on affected limb	5				
	Other - Mixed	Netz et al. 2007 [50]	SS improved reach and ability to stand and walk.	4	High	Weak +	Yellow- MEASURE	30 mins 3–5 × wk
	SCI	Bohannon [35]	Improved transfers	5	Low	Weak +	Yellow - Measure	30 mins 5 × wk
		Dunn [38]	68 % report improved ADL. Independence	5				
		Eng [22]	16/38 improved self-care	5				
Bone mineral density	SCI	Alekna [29]	SS greater BMD	3	Low to moderate	Weak +	Yellow- Measure	60 mins daily
		deBruin [37]	Little or no bone loss versus controls	3				
		Goemaere [42]	Better-preserved femoral shaft and L3 BMD	4				
		Goktepe [43]	Slight tendency to higher t-scores	4				
Strength	SCI	Edwards [39]	SS increased EMG activity	4	Low	Weak +	Yellow-Measure	30 mins, 4–5 × wk
	Other - Mixed	Netz [50]	SS increased strength	4	High	Weak +	Yellow-Measure	30 mins 3–5 × wk
Spasticity	Other - MS	Baker [32]	Downward trend for Ashworth scores (knee flex/ankle DF), reduction in spasms	2	Moderate	Weak +	Yellow-Measure	60 mins 2 × wk
	SCI	Adams [28]	Decreased extensor spasms	2	Low	Weak +	Yellow-Measure	30 mins 5 × wk
		Odeen [51]	SS reduced resistance to passive movement	4				
		Bohannon [35]	Decreased spasms	5				
		Eng [20]	9/38 reduced muscle spasms	5				
		Shields [54]	Decreased spasms	5				
		Dunn [38]	42 % report decreased spasticity	5				
		Walter [22]	Spasticity decreased	5				
Skin	SCI	Cotie [36]	Decreased temperature at 2/6 sites and decreased reactivity	2	Low to Moderate	Weak +	Yellow - Measure	30 mins 5 × wk
		Dunn [38]	17 % report decreased bed sores	5				
		Eng [20]	14/38 increased skin integrity	5				
		Walter [22]	Bed sores decreased	5				
Cardio-respiratory	Stroke	Kuznetsov [46]	52 % static standing orthostatic hypotension	2	Low	Weak -	Yellow - Measure	20–30 mins daily
	ABI	Taveggio	Static standing worsened orthostatic hypotension in 3 out of 4	2	Low	Weak -	Yellow - Measure	30 mins 3 × wk
	SCI	Edwards [39]	SS increased HR	4	Very low	Weak +	Yellow - Measure	30 mins 5 × wk
		Eng [20]	16/38 decreased swelling in legs and feet	5				
Mental	SCI/MS SCI	Kunkel [45]	67 % ‘felt better’	4	Very low	Weak +	Yellow - Measure	60 mins 4–6 × wk
		Dunn [38]	69 % increased QOL	5				
		Eng [20]	33/38 increased well-being	5				
		Walter [22]	QOL increased	5				
Pain	TBI	Richardson [52]	Time standing before experiencing pain increased	4	Very low	Weak +	Yellow - Measure	30 mins, 3–6 × wk
	SCI	Dunn [38]	Report decreased leg or back pain	5	Very low	Weak +	Yellow - Measure	30 mins 5 × wk
		Eng [20]	12/38 decreased pain	5				
Bowel	Other - Mixed	Netz [50]	SS improvement in sphincter control	4	High	Weak +	Yellow - Measure	30 mins, 3–5 × wk
	SCI	Kwok	8/17 reported improved bowel function -no difference on objective measures	2	Very low	Weak +	Yellow-Measure	30 mins 5 × wk
		Hoenig [44]	SS increased frequency and decreased bowel care time	5				
		Dunn [38]	23 % increase regularity	5				
		Eng [20]	20/38 improved bowel function	5				
		Shields [54]	Improved bowel function	5				
		Walter [22]	Improved regularity	5				
Urinary	SCI	Eng [20]	20/38 improved bladder function	5	Very low	Weak +	Yellow - Measure	30 mins 5 × wk
		Dunn [38]	21 % report decreased infections and increased ability to empty bladder	5				
		Walter [22]	Infections decreased and ability to empty bladder increased	5				

### Body structure & function outcomes

#### Range of motion

In one high quality randomized controlled trial [53], standing was more effective than no treatment and as effective as night-time splinting in preventing ankle contractures in subjects with stroke. Longitudinal cohort evidence suggests that daily standing can eliminate plantar flexion contracture in adults with acquired brain injury [55] and case-study evidence also supports this outcome with the same population [52]. A small randomized trial found that adults with secondary progressive MS showed statistically significant improvement of hip and ankle ROM over the control (exercise) group [32]. Randomized control trial [33] and case-series evidence [34] support increase in ankle ROM and, in surveys, adults with SCI describe increased leg ROM [20, 22, 38]. However, standing appears less effective in changing ROM in those with long-standing contracture [45].

#### Bone mineral density

This outcome has only been studied in the SCI population with descriptive evidence providing the strongest support for positive benefits, particularly for higher dose standing, started early and continued in the long-term. One cross-sectional study reported significantly higher BMD in the proximal femur and lumbar spine with highest BMD at proximal femur in those standing using long-leg braces [42]. Another [43] found that standing for more than 7 h a week slightly increased BMD, while standing for less than 7 h a week did not. Longitudinal cohort studies found that those standing daily for at least 1 h per day, had significantly higher BMD in the lower extremities after 2 years in comparison to those who did not stand [29] and that beginning weight-bearing immediately following SCI, decreased expected rate of BMD loss [37]. However, this may only be effective for some individuals [41]. Randomized trial evidence found that functional electrical stimulation cycling was not better than standing at retaining BMD [40] and when one leg was used as the control, and the other leg was placed on a foam wedge, there was a slight increase in the femur BMD in the “intervention” leg [33]. The foam did not appear to be compressed and the subject’s pelvis remained level, suggesting that the intervention leg was not fully loaded. However, in veterans with SCI many years after initial injury, standing did not improve BMD [45].

#### Strength and spasticity

In two case-series designs, adults in a nursing home [50] and subjects with chronic SCI [39] who performed exercises in standing devices demonstrated increased strength. However, in a large randomized trial, subjects with stroke gained more strength following robotic stepping combined with functional electrical stimulation when compared to tilt-table standing alone [46]. Two additional RCT’s including subjects with stroke [57, 58] also demonstrated that muscle strength increased more when task-specific training was added to a tilt-table intervention than standing alone. Impact of standing on spasticity or muscle spasms has only been studied in the SCI and MS populations. In a randomized cross-over study, standing decreased extensor spasms in adults with SCI more so than body weight support treadmill training however, the treadmill training group showed more decrease in flexor spasm [28] In a case-series, subjects with SCI stood on a tilt-table with a dorsiflexion wedge (15°), and had a decrease in plantar flexor spasticity [51] Standing decreased spasticity in subjects with chronic SCI in two single-case studies [35, 54] but in one [35], this decrease only lasted until the next morning. Flexor spasms at the knee and ankle showed a downward trend after standing in a randomized cross-over involving six subjects with MS [32]. In one of the highest dosage studies in this review, standing did not result in change in reflexes, tone or clonus in a case-series of six subjects with long-standing SCI or MS [45].

#### Skin

Increased resting skin temperature and decreased skin temperature reactivity have been linked to development of pressure sores. In subjects with SCI, a single session of standing resulted in temperature decreases at two sites as well as altered reactivity of skin temperature at all sites except the right calf [36]. Surveys of adults with SCI suggest that supported standing may help decrease incidence of pressure ulcers [20, 22, 38].

#### Cardio-respiratory function

A stander that enabled patients with SCI to move their trunks and perform supported exercises while standing, resulted in a positive increase in heart rate [39]. Two surveys of adults with chronic SCI who used standing devices regularly reported improved circulation and decreased edema [20, 38]. Negative side effects such as orthostatic hypotension may be problematic and may be alleviated by addition of functional electrical stimulation or stepping in the sub-acute stroke population [46]. Robotic stepping has also been shown to alleviate orthostatic hypotension in minimally conscious subjects following acquired brain injury [60].

#### Mental function and pain

A follow-up interview of adults with chronic SCI or MS, showed that 67 % continued to stand and felt healthier because of it. This suggests a positive psychological impact [45] despite lack of evidence for impact on other functions. Surveys of adults with chronic SCI also reported an increase in subjective sense of well-being or quality of life [20, 22, 38]. However, adults with SCI who participated in body weight support treadmill training reported more improvement in quality of life than those who used a standing frame [28] and a study of adults with severe stroke did not measure improvement on the Hospital Depression and Anxiety Scale [31]. Richardson [52] reported decreased pain following a standing program in an adult with traumatic brain injury. Adults with SCI also reported some reduction in pain following supported standing [20, 38].

#### Bladder and bowel function

Residents of a nursing home with a variety of neurological diagnoses who stood and exercised regularly in a standing box, showed statistically significant improvement in their anal wink reflex [50]. Other evidence for impact of standing on bowel and bladder function has only been studied with the SCI population. A randomized trial [59] found no change in objective measures of bowel function although 8/17 participants reported improvement. Survey and single case study evidence suggests that use of a standing device can improve bowel function [20, 22, 44, 54] and Dunn [38] found a correlation between this outcome and use of a standing device daily, for more than 30 min per bout. Survey data also suggests improved bladder function and decreased incidence of urinary tract infections [20, 22, 38], however, no correlation was found between number of infections and higher dosage of standing [38].

### Activity and participation outcomes

A positive trend for gross motor function, trunk control and significant improvement in balance for individuals with stroke was found following standing intervention [30]. Yet a similar study, also with a sub-acute stroke population, did not show this benefit [31]. Two randomized trials in individuals with sub-acute stroke [57, 58] suggest that adding task-specific training to tilt-table standing is more beneficial in improving gait and functional activities than supported standing alone. Two randomized trials [47, 56] and a single case study [48] found that adding biofeedback to a standing program made a significant difference in static standing balance in adults with stroke or traumatic brain injury. A mixed population study [50] found statistically significantly improved reach and ability to stand and walk, as well as a trend towards improved transfers. Survey evidence supports impact of standing devices on self-care [20], ability to carry out daily living activities, gain and maintain employment as well as promotion of ‘freedom and independence’ [38] for those with chronic SCI. Standing reportedly made transfers easier for a subject with chronic SCI, but the benefits only lasted until the next morning [35]. Body weight support treadmill training may have more impact on mobility level than supported standing alone for the SCI population [28].

## Discussion

Moderate to high quality evidence supports the positive impact of standing on ROM and activity for adults with neurological conditions. The strongest evidence, resulting from level II moderate or high quality studies, supports impact on ROM for adults with stroke and SCI. Strong evidence from a high quality randomized study, and other lower quality studies, also support the benefit of supported standing on activity outcomes such as standing symmetry and ability to maintain a stable standing position for the sub-acute and chronic stroke population. Strong evidence also supports the addition of task-specific training to tilt-table standing for improvement in gait, functional activity and muscle strength in the sub-acute stroke population.

Evidence supporting impact on ROM for the sub-acute SCI population is supported by moderate quality level II evidence as well as lower quality studies. However, evidence supporting impact on activity outcomes such as activities of daily living, independence and transfers is merely supported by case study or survey evidence. One study including those with long-standing SCI or MS [45] stands out because there were no changes in spasticity, ROM or BMD, perhaps due to the chronic nature of these factors in participants.

Evidence for impact on BMD is somewhat mixed with descriptive evidence mainly suggesting benefits for early initiation of higher-dose standing programs. There is conflicting evidence however, with one longitudinal study suggesting benefits for only some participants [41]. A weakness in all studies investigating BMD was lack of established load and may explain the varied results. Another consideration is that using a tray to support the arms may decrease ground reaction force by up to 10 % [65]. From included studies, 60 min 5–6 times a week may be a high enough dose to have a beneficial impact on BMD, while 30 min 3–6 times a week was not.

Low evidence level intervention studies support improvements in muscle strength and spasticity/tone. Adult user input and expert opinion support impact on mental function, pain and sensory, cardiopulmonary and respiratory, bowel, urinary, and skin function. Older studies suggest that standing can increase bladder pressure [66] and decrease residual volume [17] possibly improving bladder emptying. Improvements in kidney drainage [67] and reduction in renal calculi [68] suggest a possible link between standing and bone loss. While very weak quality evidence [36] suggests a positive effect on skin function, supported standing has been shown to off-load and unweight the ischial tuberosities [69].

Contradictory evidence was found regarding impact on cardio-respiratory function with orthostatic hypotension being a problem for those with SCI [70]. Frequent bouts of shorter duration appear to increase tolerance over time [71]. The addition of functional electrical stimulation [72–75], and/or passive reciprocal stepping/cycling [60, 76–78] to a standing device can ameliorate decreases in blood pressure, hypotension, autonomic dysreflexia and even mirror cardiopulmonary responses seen in active exercise [39].

A number of higher-level intervention designs were identified, addressing activity, spasticity and muscle tone, strength, BMD and ROM outcomes. Eight level II studies [30, 31, 46, 47, 53, 56–58] included a sub-acute stroke population, but only positive impact on ROM and activity was demonstrated for supported standing alone. Only one of these can be considered a high quality study [53]. No group study addressed use of standing in a chronic stroke population. Two level II studies [33, 40] included a sub-acute SCI population, and two additional level II studies [28, 36] included a chronic SCI population but there were bias concerns and risks and none was considered high quality. The remaining level II study [32] was moderate quality but only included 6 individuals with chronic MS.

Only two other systematic reviews on use of passive standing were identified in the search [12, 13]. Glickman et al. [12] included pediatric and adult subjects and, although lacking a quality rating, found adequate evidence to support positive effect on BMD, ROM, spasticity and bowel function. Newman and Barker [13] focused on higher-level intervention designs and did not include mental, cardio-respiratory, urinary, digestive/bowel, muscle strength or skin function. They concluded that weak evidence supported the effectiveness of higher dose standing on BMD and minimal ROM gains. They used the same type of risk-of-bias rating but rated one study [33] high quality whereas potential for performance bias merits down-rating. Detection bias was identified in another study [51].

This review was limited by the complexities of the electronic search. Terms such as stander or standing generate a high number of citations that are difficult to narrow down. Studies published in other languages or grey literature may have been missed. This review covered a long period of time (over 30 years) where reporting standards have changed, and some studies lacked detail about the intervention making it challenging to compare studies. Unfortunately, the bulk of studies identified achieved low-quality ratings and also included low numbers of participants resulting in low strength of recommendation. The low evidence level and disparate populations limited ability to combine results and to draw strong conclusions.

However, this review does help to establish the current evidence level, adds strength of recommendation and identifies dosage guidelines for different populations and specific ICF components. The strongest evidence supports impact on ROM and activity with SCI and stroke populations. Low evidence level studies support improvements in BMD, strength and spasticity. Adult user input and expert opinion support improvements in mental, pain and sensory, cardiopulmonary and respiratory, bowel, urinary, and skin function. Overall little information on dosage was provided, the majority of articles lacked specifics about how the standing program was implemented and no study measured actual weight bearing or muscle activity. Future research studies may benefit from use of the TIDieR checklist [79] to ensure better reporting of intervention detail, making it easier to compare results across studies.

While additional high-quality research studies would be beneficial for all outcomes, the need is particularly high for the majority of body structure and function outcomes, in particular BMD, cardio-respiratory, pain, skin, bowel and bladder function. The largest number of high-level studies was completed with sub-acute stroke patients and yet evidence for effectiveness for most outcomes is limited. Further high-level and longer-term research is warranted with this population in particular. Although there has been an extensive amount of cross-sectional and observational research conducted with the sub-acute and chronic SCI population, stronger intervention research is also warranted.

There was a notable disconnect between the qualitative and quantitative data identified in this review. In one study, no change was found on the objective measures, while a significant proportion of subjects reported an improvement in bowel function [59]. While some studies may not have used a high enough dosage of standing [41], others may have used outcome measures that were not sensitive or appropriate [59]. The evidence and quality rating used in this systematic review weighs the quantitative evidence over the qualitative, but we would be remiss to ignore subjects who consistently report that standing results in psychological, bowel and circulatory benefits that have not yet been measured by researchers. This suggests that clinicians should consult their patients about desired goals and monitor that these results are being achieved through use of qualitative, subjective or self-report in addition to objective assessments.

Future research studies should explore optimal angle of standing, possible benefits of abduction and type of stander. For adults who are dependent for transfers, standing programs require considerable time and resource commitment. Lack of attendant help has been cited as a reason for discontinuing standing [45]. Use of standing devices that facilitate transfers, are powered or built into wheelchairs may facilitate use. Many adults reported using standers in multiple short bouts (10–15 min) yet there were no quantitative studies that used this dosage parameter.

## Conclusion

Stronger evidence underpins the impact of supported standing programs on ROM and activity for stroke and SCI populations with mixed evidence supporting impact on BMD. Evidence for other outcomes is weak or very weak. Dosage data suggests that use of a standing device should occur for 30 min 5 times a week for positive impact on most outcomes such as self-care and standing balance, ROM, cardio-respiratory, strength, spasticity, pain, skin and bladder and bowel function while 60 min 4–6 times a week may be required for positive impact on BMD and mental function. While therapists can recommend with some confidence the use of a supported standing intervention to impact on ROM and activity outcomes, the evidence is less certain for other outcomes. Outcomes should be measured to ensure effectiveness for individual clients.

## Additional files

Additional file 1:
**Search strategy.** (DOCX 90 kb) Additional file 2:
**Domain based risk of bias for included primary studies.** (DOCX 134 kb) Additional file 3:
**Details of excluded studies with reasons.** (DOCX 88 kb)

## Abbreviations

BMD: Bone mineral density
GRADE: Grading of recommendations, assessment, development and evaluation
ICF: International Classification of Functioning, Disability & Health
MS: Multiple sclerosis
ROM: Range of motion
SCI: Spinal cord injury
