# Supplementary material for: Systematic review and clinical recommendations for dosage of supported home-based standing programs for adults with stroke, spinal cord injury and other neurological conditions
Source: BMC Musculoskelet Disord. 2015 Nov 17;16:358. doi: 10.1186/s12891-015-0813-x (PMC4650310; doi:10.1186/s12891-015-0813-x)
Supplement: Additional file 1: — Search strategy. (DOCX 90 kb) [file 12891_2015_813_MOESM1_ESM.docx]

**Appendix 1: Search strategy**

| **Date** | **Database** | **Search terms** | **limits** | **# articles retrieved** | **Articles saved** |
| --- | --- | --- | --- | --- | --- |
| 26th September 2015 | Ebsco Medline and Cinahl | Stander | All adult | 11 | 0 |
| “ | “ | ‘standing’ and ‘assistive technology’ | “ | 34 | 7 |
| “ | “ | ‘tilt table’ and ‘rehabilitation’ | “ | 64 | 20 |
| “ | “ | ‘standing frame’ | “ | 34 | 11 |
| “ | “ | ‘standing wheelchair’ | “ | 19 | 5 |
| “ | “ | “supported standing” | “ | 66 | 5 |
| “ | “ | ‘standing’ and ‘tilt table’ | “ | 157 | 14 |
| “ | “ | ‘standing position’ and ‘rehabilitation | “ | 196 | 2 |
| “ | “ | ‘standing’ and ‘SCI’ | “ | 141 | 23 |
| “ | “ | ‘standing equipment’ and ‘stroke’ | “ | 28 | 3 |
| 4^th^ Jan 2014 | Ovid EBM reviews | stander | No limits | 6 | 0 |
| “ | “ | standing AND assistive technology’ | “ | 7 | 0 |
| “ | “ | Tilt-table AND rehabilitation | “ | 14 | 5 |
| “ | “ | standing frame | “ | 12 | 9 |
| “ | “ | standing wheelchair | “ | 1 | 0 |
| “ | “ | supported standing | “ | 10 | 6 |
| “ | Ovid Embase | Stander AND therapy | “ | 55 | 0 |
| “ | “ | standing AND assistive technology’ | “ | 48 | 5 |
| “ | “ | standing frame | “ | 66 | 14 |
| “ | “ | standing wheelchair | “ | 12 | 9 |
| “ | “ | supported standing | “ | 47 | 7 |

All results from electronic search imported into refworks. Total references imported = 440

Duplicates removed, total articles from electronic search = 314

Articles retrieved through manual searching = 72

Total articles reviewed by title and abstract = 386

Articles agreed by both authors to be reviewed full text = 74
