# Supplementary material for: Systematic review and clinical recommendations for dosage of supported home-based standing programs for adults with stroke, spinal cord injury and other neurological conditions
Source: BMC Musculoskelet Disord. 2015 Nov 17;16:358. doi: 10.1186/s12891-015-0813-x (PMC4650310; doi:10.1186/s12891-015-0813-x)
Supplement: Additional file 2: — Domain based risk of bias for included primary studies. (DOCX 134 kb) [file 12891_2015_813_MOESM2_ESM.docx]

**Appendix 2:** **Domain based risk of bias for included primary studies**

| **Study** | **Selection bias** | **Performance bias** | **Attrition bias** | **Detection bias** | **Reporting bias** | **Overall risk of bias** |
| --- | --- | --- | --- | --- | --- | --- |
| **Adams [28]** | Moderate risk | Low risk | Low risk | Serious risk | Low risk | Moderate risk |
| **Alekna [29]** | Serious risk | Low risk | Low risk | Low risk | Low risk | Serious risk |
| **Allison [30]** | Low risk | Moderate risk | Moderate risk | Low risk | Low risk | Moderate risk |
| **Bagley [31]** | Low risk | Moderate risk | Moderate risk | Low risk | Low risk | Moderate risk |
| **Baker [32]** | Moderate risk | Moderate risk | Low risk | Low risk | Low risk | Moderate risk |
| **Ben [33]** | Low risk | Moderate risk | Low risk | Low risk | Low risk | Moderate risk |
| **Bohannon [34]** | Low risk | Low risk | Low risk | Serious risk | Low risk | Serious risk |
| **Cotie[36]** | Moderate risk | Low risk | Low risk | Moderate risk | Low risk | Moderate risk |
| **De Bruin [37]** | Serious risk | Serious risk | Moderate risk | Low risk | Unclear risk | Serious risk |
| **Edwards [39]** | Unclear risk | Low risk | Low risk | Serious risk | Low risk | Serious risk |
| **Eser [40]** | Serious risk | Low risk | Low risk | Low risk | Low risk | Serious risk |
| **Frey-Rindova [41]** | Serious risk | Low risk | Low risk | Low risk | Low risk | Serious risk |
| **Goemaere [42]** | Low risk | Moderate risk | Low risk | Low risk | Low risk | Moderate risk |
| **Goktepe [43]** | Moderate risk | Moderate risk | Low risk | Low risk | Low risk | Moderate risk |
| **Kim [57]** | Low risk | Moderate risk | Low risk | Low risk | Low risk | Moderate risk |
| **Kim [58]** | Low risk | Moderate risk | Low risk | Low risk | Low risk | Moderate risk |
| **Kunkel [45]** | Low risk | Moderate risk | Low risk | Serious risk | Low risk | Serious risk |
| **Kuznetsov [46]** | Serious risk | Low risk | Moderate risk | Moderate risk | Low risk | Serious risk |
| **Kwok [59]** | Moderate risk | Moderate risk | Low risk | Moderate risk | Low risk | Moderate risk |
| **Lee [47]** | Unclear risk | Unclear risk | Low risk | Serious risk | Low risk | Serious risk |
| **Nelson[49]** | NA | Low risk | Low risk | Low risk | Low risk | Low risk |
| **Netz [50]** | Low risk | Low risk | Low risk | Low risk | Low risk | Low risk |
| **Odeen [51]** | Unclear risk | Low risk | Low risk | Serious risk | Low risk | Serious risk |
| **Richardson [52]** | N/A | Low risk | Low risk | Serious risk | Low risk | Serious risk |
| **Robinson [53]** | Low risk | Low risk | Low risk | Low risk | Low risk | Low risk |
| **Singer [54]** | Unclear risk | Low risk | Low risk | Low risk | Low risk | Unclear risk |
| **Taveggia [60]** | Low risk | Low risk | Moderate risk | Moderate risk | Low risk | Moderate risk |
| **Wong [56]** | Unclear risk | Unclear risk | Low risk | Serious risk – | Low risk | Serious risk |
