# Supplementary material for: Systematic review and clinical recommendations for dosage of supported home-based standing programs for adults with stroke, spinal cord injury and other neurological conditions
Source: BMC Musculoskelet Disord. 2015 Nov 17;16:358. doi: 10.1186/s12891-015-0813-x (PMC4650310; doi:10.1186/s12891-015-0813-x)
Supplement: Additional file 3: — Details of excluded studies with reasons. (DOCX 88 kb) [file 12891_2015_813_MOESM3_ESM.docx]

**Appendix 3:** Details of excluded studies with reasons

Two reviews were excluded as being narrative reviews [1,4] and three were excluded as having mixed interventions.[73-75] One review and two surveys were excluded as having mixed population.[9,76,77] In addition, two descriptive studies were excluded as lacking sufficient detail on outcomes [61,78] and five studies were non-peer reviewed.[18,54-57]

Fifteen studies did not meet our criteria for a passive standing intervention: two of these did not include a passive standing intervention;[79,80,94] one did not use a relevant piece of standing equipment;[81] three did not use the minimum standing angle;[82-84] and nine did not include a sufficient length of time or number of sessions to meet inclusion criteria.[62,65-68,85-88,95] Three studies did not meet the criteria for population.[89-91] Two studies [63,92] and one review [10] did not include sufficient dosage information and one study was not about standing outcomes.[93]

**References:**

1. Arva J, Paleg G, Lange M, et al. RESNA position on the application of wheelchair standing devices. *Assist Technol*. 2009;21(3):161-168; quiz 169-171. doi:10.1080/10400430903175622.

2. World Health Organization. *International Classification of Functioning, Disability & Health (ICF)*. Geneva, Switzerland; 2001.

3. Sackley C, Brittle N, Patel S, et al. The prevalence of joint contractures, pressure sores, painful shoulder, other pain, falls, and depression in the year after a severely disabling stroke. *Stroke*. 2008;39(12):3329-3334. doi:10.1161/STROKEAHA.108.518563.

4. Giangregorio L, McCartney N. Bone loss and muscle atrophy in spinal cord injury: epidemiology, fracture prediction, and rehabilitation strategies. *J Spinal Cord Med*. 2006;29(5):489-500. http://www.pubmedcentral.nih.gov/articlerender.fcgi?artid=1949032&tool=pmcentrez&rendertype=abstract.

5. Hoang PD, Gandevia SC, Herbert RD. Prevalence of joint contractures and muscle weakness in people with multiple sclerosis. *Disabil Rehabil*. 2013:1-6. doi:10.3109/09638288.2013.854841.

6. Fergusson D, Hutton B, Drodge A. The epidemiology of major joint contractures: a systematic review of the literature. *Clin Orthop Relat Res*. 2007;456(456):22-29. doi:10.1097/BLO.0b013e3180308456.

7. Kwah LK, Harvey L a, Diong JHL, Herbert RD. Half of the adults who present to hospital with stroke develop at least one contracture within six months: an observational study. *J Physiother*. 2012;58(1):41-47. doi:10.1016/S1836-9553(12)70071-1.

8. Singer BJ, Jegasothy GM, Singer KP, Allison GT, Dunne JW. Incidence of ankle contracture after moderate to severe acquired brain injury. *Arch Phys Med Rehabil*. 2004;85(9):1465-1469. doi:10.1016/j.apmr.2003.08.103.

9. Glickman L, Geigle P, Paleg G. A systematic review of supported standing programs. *J Pediatr Rehabil Med*. 2010;3(3):197-213.

10. Newman M, Barker K. The effect of supported standing in adults with upper motor neurone disorders: a systematic review. *Clin Rehabil*. 2012;26(12):1059-1077. doi:10.1177/0269215512443373.

11. Abramson A, Delagi E. Influence of weight-bearing and muscle contraction on disuse osteoporosis. *Arch Phys Med Rehabil*. 1961;(March):147-151. http://europepmc.org/abstract/MED/13681127. Accessed April 21, 2014.

12. Climo S. The erect position as an aid in the care of the paraplegic. *Plast Reconstr Surg*. 1954;13(1):65-69.

13. Kim K. The Kim self-stander for wheelchair patients. *Arch Phys Med Rehabil*. 1961;42:599-601.

14. Machek O. A new standing table. *Am J Occup Ther*. 1955;9(4):158-163.

15. Rogers E. The care of paraplegic patients in general hospitals. *Can Med Assoc J*. 1948;59(8):338-343. http://www.pubmedcentral.nih.gov/articlerender.fcgi?artid=1591168&tool=pmcentrez&rendertype=abstract.

16. Willhite C. The quadriplegic standing frame. *Arch Phys Med Rehabil*. 1954;35(4):236-239.

17. Eng J, Levins S, Townson A, Mah-Jones D, Bremner J, Huston G. Use of prolonged standing for individuals with spinal cord injuries. *Phys Ther*. 2001;81(8):1392-1399. http://physther.net/content/81/8/1392.short. Accessed December 31, 2012.

18. Warren B, Brewer J, Herrara E, Perkash I. The frequency of standing frame use in a spinal cord injured outpatient population. In: *American Corrective Therapy Association National Conference, New York*. Palo Alto, CA: VAMC; 1985.

19. Walter J, Sola P, J S, Lucero Y, Langbein E, Weaver F. Implications for a home standing program for individuals with spinal cord injury. *J Spinal Cord Med*. 1999;22(3):152-158.

20. Moher D, Liberati A, Tetzlaff J, Altman D. The PrefeReporting Items for Systematic Reviews and Meta-Analyses: The PRISMA Statement. *PLoS Med*. 2009;6(6):e1000097.

21. Higgins J, Green S, eds. *Cochrane Handbook for Systematic Reviews of Interventions 5.1.0*. The Cochrane Collaboration; 2011:187-241.

22. OCEBM Levels of Evidence Working Group. The Oxford Levels of Evidence 2. *Oxford Cent Evid Based Med*. 2011;1. www.cebm.net/index.aspx?o=5653. Accessed March 1, 2014.

23. Guyatt G, Oxman AD, Akl EA, et al. GRADE guidelines: 1. Introduction-GRADE evidence profiles and summary of findings tables. *J Clin Epidemiol*. 2011;64(4):383-394. doi:10.1016/j.jclinepi.2010.04.026.

24. Novak I. Evidence to practice commentary: the evidence alert traffic light grading system. *Phys Occup Ther Pediatr*. 2012;32(3):256-259. doi:10.3109/01942638.2012.698148.

25. Adams MM, Hicks AL. Comparison of the effects of body-weight-supported treadmill training and tilt-table standing on spasticity in individuals with chronic spinal cord injury. *J Spinal Cord Med*. 2011;34(5):488-494. doi:10.1179/2045772311Y.0000000028.

26. Alekna V, Tamulaitiene M, Sinevicius T, Juocevicius A. Effect of weight-bearing activities on bone mineral density in spinal cord injured patients during the period of the first two years. *Spinal Cord*. 2008;46(11):727-732. doi:10.1038/sc.2008.36.

27. Allison R, Dennett R. Pilot randomized controlled trial to assess the impact of additional supported standing practice on functional ability post stroke. *Clin Rehabil*. 2007;21(7):614-619. doi:10.1177/0269215507077364.

28. Bagley P, M H, Forster A, Smith J, Young J. A randomized trial evaluation of the Oswestry Standing Frame for patients after stroke. *Clin Rehabil*. 2005;19:354-364.

29. Baker K, Cassidy E, Rone-Adams S. Therapeutic standing for people with multiple sclerosis: Efficacy and feasibility. *Int J Ther Rehabil*. 2007;14(3):104-109.

30. Ben M, Harvey L, Denis S, et al. Does 12 weeks of regular standing prevent loss of ankle mobility and bone mineral density in people with recent spinal cord injuries ? *Aust J Physiother*. 2001;51:251-256.

31. Bohannon R, Larkin P. Passive Ankle Dorsiflexion Increases in Patients After a Regimen of Tilt Table-Wedge Board Standing A Clinical Report. *Phys Ther*. 1985;65(11):1676-1678. http://physther.net/content/65/11/1676.short. Accessed January 2, 2013.

32. Bohannon R. Tilt table standing for reducing spasticity after spinal cord injury. *Arch Phys Med Rehabil*. 1993;74:1121-1122.

33. Cotie LM, Geurts CLM, Adams MME, MacDonald MJ. Leg skin temperature with body-weight-supported treadmill and tilt-table standing training after spinal cord injury. *Spinal Cord*. 2010;49(1):149-153. doi:10.1038/sc.2010.52.

34. De Bruin ED, Frey-rindova P, Herzog RE, et al. Changes of Tibia Bone Properties After Spinal Cord Injury : Effects of Early Intervention. *Arch Phys Med Rehabil*. 1999;80(February):214-220.

35. Dunn R, Walter J, Lucero Y. Follow-up assessment of standing mobility device users. *Assist Technol*. 1998;10:84-93. http://www.tandfonline.com/doi/abs/10.1080/10400435.1998.10131966. Accessed December 31, 2012.

36. Edwards LC, Layne CS. Effect of dynamic weight bearing on neuromuscular activation after spinal cord injury. *Am J Phys Med Rehabil*. 2007;86(6):499-506. doi:10.1097/PHM.0b013e31805b764b.

37. Eser P, de Bruin ED, Telley I, Lechner HE, Knecht H, Stüssi E. Effect of electrical stimulation-induced cycling on bone mineral density in spinal cord-injured patients. *Eur J Clin Invest*. 2003;33(5):412-419. http://www.ncbi.nlm.nih.gov/pubmed/12713456.

38. Frey-Rindova P, Bruin E De, Stüssi E, Dumbacher M, Dietz V. Bone mineral density in upper and lower extremities during 12 months after spinal cord injury measured by peripheral quantitative computed tomography. *Spinal Cord*. 2000;38:26-32. http://ukpmc.ac.uk/abstract/MED/10762194. Accessed December 31, 2012.

39. Goemaere S, Laere M Van. Bone mineral status in paraplegic patients who do or do not perform standing. *Osteoporos Int*. 1994;4:138-143. http://www.springerlink.com/index/X72N6T6G5L18G0LQ.pdf. Accessed December 31, 2012.

40. Goktepe A, Tugcu I, Yilmaz B. Does standing protect bone density in patients with chronic spinal cord injury. *J Spinal Cord Med*. 2008;31:197-201. http://www.ncbi.nlm.nih.gov/pmc/articles/PMC2565474/. Accessed January 1, 2013.

41. Hoenig H, Murphy T. Case study to evaluate a standing table for managing constipation. *SCI Nurs*. 2001;18(2):74-77. http://ukpmc.ac.uk/abstract/MED/12035465. Accessed January 2, 2013.

42. Kunkel C, Scremin A, Eisenberg B, Garcia J, Roberts S, Martinez S. Effect of“ standing” on spasticity, contracture, and osteoporosis in paralyzed males. *Arch Phys Med Rehabil*. 1993;74:73-78. http://ukpmc.ac.uk/abstract/MED/8420525. Accessed January 2, 2013.

43. Kuznetsov AN, Rybalko N V, Daminov VD, Luft AR. Early poststroke rehabilitation using a robotic tilt-table stepper and functional electrical stimulation. *Stroke Res Treat*. 2013;2013(Article ID 946056):1-9. doi:10.1155/2013/946056.

44. Lee M, Wong M, Tang F. Clinical evaluation of a new biofeedback standing balance training device. *J Med Eng*. 1996;20(2):60-66. http://informahealthcare.com/doi/abs/10.3109/03091909609008381. Accessed February 27, 2013.

45. Matjacić Z, Hesse S, Sinkjaer T. BalanceReTrainer: a new standing-balance training apparatus and methods applied to a chronic hemiparetic subject with a neglect syndrome. *NeuroRehabilitation*. 2003;18(3):251-259. http://www.ncbi.nlm.nih.gov/pubmed/14530590.

46. Nelson D, Schau E. Effects of a standing table on work productivity and posture in an adult with developmental disabilities. *Work*. 1997;9:13-20. http://www.ingentaconnect.com/content/els/10519815/1997/00000009/00000001/art00019. Accessed February 4, 2013.

47. Netz Y, Argov E, Burstin A, et al. Use of a device to support standing during a physical activity program to improve function of individuals with disabilities who reside in a nursing home. *Disabil Rehabil Assist Technol*. 2007;2(1):43-49. doi:10.1080/17483100601143371.

48. Odeen I, Knutsson E. Evaluation of the effects of muscle stretch and weight load in patients with spastic paraplegia. *Scand J Rehabil Med*. 1981;13(4):117-121. http://ukpmc.ac.uk/abstract/MED/7347432. Accessed March 4, 2013.

49. Richardson D. The use of the tilt-table to effect passive tendo-achilles stretch in a patient with head injury. *Physiother Theory Pract*. 1991;7:45-50.

50. Robinson W, Smith R, Aung O, Ada L. No difference between wearing a night splint and standing on a tilt table in preventing ankle contracture early after stroke: a randomised trial. *Aust J Physiother*. 2008;54:33-38. http://svc019.wic048p.server-web.com/ajp/vol_54/1/AustJPhysiotherv54i1Robinson.pdf. Accessed December 31, 2012.

51. Shields RK, Dudley-Javoroski S. Monitoring standing wheelchair use after spinal cord injury: A case report. *Disabil Rehabil*. 2005;27(3):142-146. doi:10.1080/09638280400009337.

52. Singer B, Dunne J, Singer K, Jegasothy G, Allison G. Non-surgical management of ankle contracture following acquired brain injury. *Disabil Rehabil*. 2004;26(6):335-345. doi:10.1080/0963828032000174070.

53. Wong A, Lee M. The development and clinical evaluation of a standing biofeedback trainer. *J Rehabil Res Dev*. 1997;34(3):322-327. http://www.rehab.research.va.gov/jour/97/34/3/pdf/wong.pdf. Accessed February 4, 2013.

54. Aukland K, Lombard I, Paleg G. Considerations in passive standing programs for clients who are medically fragile. *Pediatr Phys Ther*. 2004;16(1):49.

55. Hendrie W. Stand and deliver! How the use of an Owestry standing frame improved sitting balance and function in a case of secondary progressive MS. *Synapse*. 2005;Autumn/Win:20-22.

56. Biering-Sørensen F, Hansen RB, Biering-Sørensen J. Mobility aids and transport possibilities 10-45 years after spinal cord injury. *Spinal Cord*. 2004;42(12):699-706. doi:10.1038/sj.sc.3101649.

57. Yaziciotiu. The effect of tilt table therapy on pulmonary functions in tetraplegic and high level paraplegic patients. *Turkiye Fiz Ripve Rehabil Derg*. 2013;59:490.

58. Bernhardt K a, Beck L a, Lamb JL, Kaufman KR, Amin S, Wuermser L-A. Weight bearing through lower limbs in a standing frame with and without arm support and low-magnitude whole-body vibration in men and women with complete motor paraplegia. *Am J Phys Med Rehabil*. 2012;91(4):300-308. doi:10.1097/PHM.0b013e31824aab03.

59. Gould DW, Hsieh ACL, Tinckler LF, Physiol J. The effect of posture on bladder pressure. *J Physiol*. 1955;129:448-453.

60. Bakewell J. Choosing support equipment in children’s therapy. *Int J Ther Rehabil*. 2007;14(8):379-381.

61. Kreutz D. Standing frames and standing wheelchairs: Implications for standing. *Top Spinal Cord Inj Rehabil*. 2000;5(4):24-28. http://thomasland.metapress.com/index/P8YCWGEHC1VP2VC1.pdf. Accessed January 1, 2013.

62. Sprigle S, Maurer C, Soneblum SE, Sorenblum SE. Load redistribution in variable position wheelchairs in people with spinal cord injury. *J Spinal Cord Med*. 2010;33(1):58-64. http://www.pubmedcentral.nih.gov/articlerender.fcgi?artid=2853329&tool=pmcentrez&rendertype=abstract.

63. Chelvarajah R, Knight SL, Craggs MD, Middleton FR. Orthostatic hypotension following spinal cord injury: impact on the use of standing apparatus. *NeuroRehabilitation*. 2009;24(3):237-242. doi:10.3233/NRE-2009-0474.

64. Figoni S. Cardiovascular and haemodynamic responses to tilting and to standing in tetraplegic patients: a review. *Paraplegia*. 1984;22:99-109. http://www.nature.com/sc/journal/v22/n2/abs/sc198418a.html. Accessed March 7, 2013.

65. Chao CY, Cheing GL. The Effects of Lower-Extremity Functional Electric Stimulation on the Orthostatic Responses of People With Tetraplegia. *Arch Phys Med Rehabil*. 2005;86(7):1427-1433. doi:10.1016/j.apmr.2004.12.033.

66. Faghri PD, Yount JP, Pesce WJ, Seetharama S, Votto JJ. Circulatory hypokinesis and functional electric stimulation during standing in persons with spinal cord injury. *Arch Phys Med Rehabil*. 2001;82(11):1587-1595. doi:10.1053/apmr.2001.25984.

67. Faghri PD, Yount J. Electrically induced and voluntary activation of physiologic muscle pump: a comparison between spinal cord-injured and able-bodied individuals. *Clin Rehabil*. 2002;16(8):878-885. http://www.ncbi.nlm.nih.gov/pubmed/12501950.

68. Jacobs P, Johnson B, Mahoney E. Physiologic responses to electrically assisted and frame-supported standing in persons with paraplegia. *J Spinal Cord Med*. 2003;26:384-389. http://www.ncbi.nlm.nih.gov/pubmed/14992341. Accessed January 2, 2013.

69. Craven CTD, Gollee H, Coupaud S, Purcell M a, Allan DB. Investigation of robotic-assisted tilt-table therapy for early-stage spinal cord injury rehabilitation. *J Rehabil Res Dev*. 2013;50(3):367-378. http://www.ncbi.nlm.nih.gov/pubmed/23881763.

70. Luther MS, Krewer C, Müller F, Koenig E. Comparison of orthostatic reactions of patients still unconscious within the first three months of brain injury on a tilt table with and without integrated stepping. A prospective, randomized crossover pilot trial. *Clin Rehabil*. 2008;22(12):1034-1041. doi:10.1177/0269215508092821.

71. Yoshida T, Masani K, Sayenko DG, Miyatani M, Fisher J a, Popovic MR. Cardiovascular response of individuals with spinal cord injury to dynamic functional electrical stimulation under orthostatic stress. *IEEE Trans Neural Syst Rehabil Eng*. 2013;21(1):37-46. doi:10.1109/TNSRE.2012.2211894.

72. Hoffmann TC, Glasziou PP, Boutron I, et al. Better reporting of interventions: template for intervention description and replication (TIDieR) checklist and guide. *BMJ*. 2014;348(March):g1687. doi:10.1136/bmj.g1687.

73. Ashe MC, Craven C, Eng JJ, Krassioukov A. Prevention and Treatment of Bone Loss after a Spinal Cord Injury: A Systematic Review. *Top Spinal Cord Inj Rehabil*. 2007;13(1):123-145. doi:10.1310/sci1301-123.

74. Hammer A, Nilsagård Y, Wallquist M. Balance training in stroke patients – a systematic review of randomized, controlled trials. *Adv Physiother*. 2008;10(4):163-172. doi:10.1080/14038190701757656.

75. Biering-Sørensen F, Hansen B, Lee BSB. Non-pharmacological treatment and prevention of bone loss after spinal cord injury: a systematic review. *Spinal Cord*. 2009;47(7):508-518. doi:10.1038/sc.2008.177.

76. Nordström B, Näslund A, Eriksson M, Nyberg L, Ekenberg L. The Impact of Supported Standing on Well-Being and Quality of Life. *Physiother Canada*. 2013;65(4):344-352. doi:10.3138/ptc.2012-27.

77. Nordström B, Nyberg L, Ekenberg L, Näslund A. The psychosocial impact on standing devices. *Disabil Rehabil Assist Technol*. 2013:1-8. doi:10.3109/17483107.2013.807443.

78. Daniels N, Pinnington L, Clift M. A review of standing frame features and their impact on user acceptability. *Int J Ther Rehabil*. 2005;12(8):363-368. http://www.ijtr.co.uk/cgi-bin/go.pl/library/abstract.html?uid=19539. Accessed January 1, 2013.

79. Davis R, Sanborn C, Nichols D, Bazett-Jones D, Dugan E. The effects of whole body vibration on bone mineral density for a person with a spinal cord injury: a case study. *Adapt Phys Act Q*. 2010;27:60-72. http://scholarworks.boisestate.edu/kinesiology_facpubs/3/. Accessed January 1, 2013.

80. Solopova I a, Tihonova DY, Grishin a a, Ivanenko YP. Assisted leg displacements and progressive loading by a tilt table combined with FES promote gait recovery in acute stroke. *NeuroRehabilitation*. 2011;29(1):67-77. doi:10.3233/NRE-2011-0679.

81. Illman a, Stiller K, Williams M. The prevalence of orthostatic hypotension during physiotherapy treatment in patients with an acute spinal cord injury. *Spinal Cord*. 2000;38(12):741-747. http://www.ncbi.nlm.nih.gov/pubmed/11175374.

82. Kaplan P, Roden W, Gilbert E, Richards L, Goldschmidt J. Reduction of hypercalciuria in tetraplegia after weight-bearing and strengthening exercises. *Paraplegia*. 1981;19:289-293.

83. Trees DW, Ketelsen C a., Hobbs J a. Use of a Modified Tilt Table for Preambulation Strength Training as an Adjunct to Burn Rehabilitation: A Case Series. *J Burn Care Rehabil*. 2003;24(2):97-103. doi:10.1097/01.BCR.0000054172.81638.BF.

84. Trees D, Coale N. Use of a dynamic tilt-table for preambulation strength training of severely deconditioned patients. *Acute Care Perspect*. 2007;16(3):6-9.

85. Field-Fote EC, Brown KM, Lindley SD. Influence of posture and stimulus parameters on post-activation depression of the soleus H-reflex in individuals with chronic spinal cord injury. *Neurosci Lett*. 2006;410(1):37-41. doi:10.1016/j.neulet.2006.09.058.

86. Kawashima N, Sekiguchi H, Miyoshi T, Nakazawa K, Akai M. Inhibition of the human soleus Hoffman reflex during standing without descending commands. *Neurosci Lett*. 2003;345(1):41-44. doi:10.1016/S0304-3940(03)00485-3.

87. Treger I, Shafir O, Keren O, Ring H. Cerebral blood flow velocity during postural changes on tilt table in stroke patients. *Eura Medicophys*. 2005;41(4):293-296. http://www.ncbi.nlm.nih.gov/pubmed/16474283. Accessed January 12, 2014.

88. Treger I, Shafir O, Keren O, Ring H. Orthostatic hypotension and cerebral blood flow velocity in the rehabilitation of stroke patients. *Int J Rehabil Res*. 2006;29(4):339-342. doi:10.1097/MRR.0b013e328010c87d.

89. Chang AT, Boots R, Hodges PW, Paratz J. Standing with assistance of a tilt table in intensive care: a survey of Australian physiotherapy practice. *Aust J Physiother*. 2004;50(1):51-54. http://www.ncbi.nlm.nih.gov/pubmed/14987193.

90. Chang AT, Boots RJ, Hodges PW, Thomas PJ, Paratz JD. Standing with the assistance of a tilt table improves minute ventilation in chronic critically ill patients. *Arch Phys Med Rehabil*. 2004;85(12):1972-1976. doi:10.1016/j.apmr.2004.03.024.

91. Hashim AM, Joseph LH, Embong J, Kasim Z, Mohan V. Tilt table practice improved ventilation in a patient with prolonged artificial ventilation support in intensive care unit. *Iran J Med Sci*. 2012;37(1):54-57. http://www.pubmedcentral.nih.gov/articlerender.fcgi?artid=3470296&tool=pmcentrez&rendertype=abstract.

92. Nordström B, Näslund A, Ekenberg L. On an equal footing: adults’ accounts of the experience of using assistive devices for standing. *Disabil Rehabil Assist Technol*. 2013;8(1):49-57. doi:10.3109/17483107.2012.678031.

93. Gontkovsky S, Huff C. Use of cognitive-behavioral interventions to facilitate tolerance of a passive standing program in an individual with quadriparesis. *SCI Psychosoc Process*. 2005;18(3):164-169.

94. Laubacher M, Perret C, Hunt KJ. Work-rate-guided exercise testing in patients with incomplete spinal cord injury using a robotics-assisted tilt-table. *Disabil Rehabil Assist Technol*. 2014;3107(September):1-6. doi:10.3109/17483107.2014.908246.

95. Chang K-H, Liou T-H, Sung J-Y, Wang C-Y, Genant HK, Chan WP. Femoral Neck Bone Mineral Density Change Is Associated with Shift in Standing Weight in Hemiparetic Stroke Patients. *Am J Phys Med Rehabil*. 2014;93(6):477-485. doi:10.1097/PHM.0000000000000053.
